# Interpenetrating Low-Molecular Weight Hyaluronic Acid in Hyaluronic Acid-Based *In Situ* Hydrogel Scaffold for Periodontal and Oral Wound Applications

**DOI:** 10.3390/polym14224986

**Published:** 2022-11-17

**Authors:** Porniweat Rosaming, Jirakit Jirayupapong, Sirikool Thamnium, Yu Yu Win, Vudhiporn Limprasutr, Ratchanee Rodsiri, Prasit Pavasant, Jittima A. Luckanagul

**Affiliations:** 1 Department of Pharmaceutics and Industrial Pharmacy, Faculty of Pharmaceutical Sciences, Chulalongkorn University, Bangkok 10330, Thailand; 2 Department of Chemistry, University of South Florida, Tampa, FL 33620, USA; 3 Department of Pharmacology and Physiology, Faculty of Pharmaceutical Sciences, Chulalongkorn University, Bangkok 10330, Thailand; 4 Department of Anatomy, Faculty of Dentistry, Chulalongkorn University, Bangkok 10330, Thailand; 5 Center of Excellence in Natural Products for Ageing and Chronic Diseases, Chulalongkorn University, Bangkok 10330, Thailand

**Keywords:** hyaluronic acid, hydrogels, drug delivery system, tissue engineering, periodontal ligament stem cells

## Abstract

Tissues engineering has gained a lot of interest, since this approach has potential to restore lost tooth-supporting structures, which is one of the biggest challenges for periodontal treatment. In this study, we aimed to develop an *in situ* hydrogel that could conceivably support and promote the regeneration of lost periodontal tissues. The hydrogel was fabricated from methacrylated hyaluronic acid (MeHA). Fragment/short-chain hyaluronic acid (sHA) was incorporated in this hydrogel to encourage the bio-synergistic effects of two different molecular weights of hyaluronic acid. The physical properties of the hydrogel system, including gelation time, mechanical profile, swelling and degrading behavior, etc., were tested to assess the effect of incorporated sHA. Additionally, the biological properties of the hydrogels were performed in both in vitro and in vivo models. The results revealed that sHA slightly interfered with some behaviors of networking systems; however, the overall properties were not significantly changed compared to the base MeHA hydrogel. In addition, all hydrogel formulations were found to be compatible with oral tissues in both in vitro and in vivo models. Therefore, this HA-based hydrogel could be a promising delivery system for low molecular weight macromolecules. Further, this approach could be translated into the clinical applications for dental tissue regeneration.

## 1. Introduction

Periodontal or gum disease, a prevalent infectious disease worldwide, causes the damage to supporting tissues around teeth and can lead to tooth loss. Periodontal disease can be managed by controlling infections, with mechanical debridement of the tooth surface (such as scaling and root planning, curettage, flap surgery with or without bone graft, etc.), and then the loss tissues would be spontaneously restored. Nevertheless, the regeneration of the lost supporting tissues does not succeed in every case; thus, alternative treatments are needed. Periodontal tissues engineering has gained interest in recent years, as this approach provides complexity to restore lost tooth-supporting structures with inert materials. Attempts for successful tissue regeneration are ultimately dependent on the interplay among the scaffold, cells, and bioactive signals [1]. Hydrogels, a widely employed scaffold, can be used as an alternative, as they are cytocompatible, possesses distinctive qualities, and are utilized in drug-controlled release [2,3].

Hyaluronic acid (HA), a bio-polysaccharide a prominent component in the extracellular matrix of connective tissues and the periodontal ligament matrix, has the potential to achieve beneficial effects in periodontal tissue regeneration [4,5,6]. HA plays a vital role in regulating cell adherence, migration, and differentiation through various binding proteins and cell-surface receptors, such as CD44 [6]. The binding of HA fragments (below 5000 Da) to CD44 and receptors for HA-mediated motility (RHAMM) could stimulate cell cycle progression, resulting in signal transduction activation and mitogenesis [7,8,9]. However, a limitation of HA administration is its fast turned over rate in the body. HA is digested by hyaluronidase, resulting in short tissue half-lives ranging from hours to days [10]. Therefore, modification of the HA chain is needed to customize the properties of the resulting material.

Chemical modifications of three targeted functional groups (the glucuronic acid carboxylic acid, the primary and secondary hydroxyl groups, and the N-acetyl group), on an HA chain with reactive molecules (e.g., acrylates, methacrylates, maleimides) could permit crosslinking (including via Michael addition reactions or photoinitiated radical polymerizations) and generate stiffness hydrogels [4,11]. Hydrogels with proper mechanical properties would not only stabilize the networking system when placed between two distinct hard-tissue structures, but would also mimic features of the native extra cellular matrix that affects to cell behaviors [12,13]. Ibrahim and his team invented a hydrogel system containing both large and small molecular weights of hyaluronic acid. Their findings showed that the amount of HA oligomer had effect on the hydrogel’s properties, such as its swelling ratio and rheological profile. However, greater benefits on the attachment and proliferation of endothelial cells were observed. Further, the HA oligomer had a very mild ability to enhance inflammation [14]. At this time, there are few studies which have investigated the use of hydrogels containing two different molecular weights of HA as a supporting material for periodontal tissue engineering. Particularly, the effect of this system has not yet been explored in both in vitro and in vivo models. Therefore, there is a clear motivation to investigate the application of a hydrogel system that contains two different molecular weights of HA to support the regeneration of lost periodontal tissues. In this study, the impacts of incorporated short-chain HA (sHA: 3 kDa) suspended in a thiol-ene crosslinking HA hydrogel on the physical properties (e.g., gelation time, mechanical properties, swelling, and degradation profile) and biological properties were investigated.

## 2. Materials and Methods

### 2.1. Materials

Sodium hyaluronate (HA, MW 47 kDa and 3 kDa) was purchased from Liuzhou Shengqiang Biotech Co., Ltd. (Guangxi, China). Methacrylic anhydride (Me, MW 154.16 g/mol), dithiothreitol (DTT, molecular weight 153.25 g/mol), carbazole, and crystal violet were purchased from Sigma-Aldrich (St. Louis, MO, USA). Sodium tetraborate (Na_2_B_4_O_7_), deuterium oxide (D_2_O), sodium hydroxide (NaOH), absolute ethanol, and absolute methanol were from Merck (Darmstadt, Germany). Dulbecco’s Modified Eagle’s Medium (DMEM)—high glucose, trypsin-EDTA solution, L-glutamine, fetal bovine serum (FBS), and penicillin-streptomycin (10,000 U/mL) were purchased from Gibco (Waltham, MA, USA). Presto Blue ^TM^, a resazurin-based solution, was from Invitrogen (Waltham, MA, USA).

### 2.2. Polymer Synthesis and Structure Elucidation

HA (47 kDa) was modified by Me via an esterification reaction, following the previous protocol [15]. In brief, 10-fold molar excess of Me was added dropwise into 1% *w*/*v* HA solution in potassium phosphate buffer (PBS) at 0–4 °C, under basic conditions (pH 9–10, adjusted by 5 M NaOH). In this basic solution, the hydroxyl group of HA would be deprotonated, whereas the ester group of Me was cleaved. The reactive moiety of cleaved Me interacts with the deprotonated hydroxyl group of HA and generates MeHA plus methacrylic acid as a by-product. After overnight continuing reaction, the solution was purified by dialysis against ultrapure water for ≥48 h at 4 °C before removal of precipitated residues by centrifugation. The supernatant was collected, flash frozen and lyophilized for 2–3 days, then stored at −20 °C. The lyophilized polymers were dissolved in D_2_O before being analyzed with a 400 MHz ^1^H NMR spectrometer (Bruker, Rheinstetten, Germany). The D_2_O peak was calibrated until it represented 4.8 ppm. The degree of modification was calculated by comparing the integrals of the peaks of the protons on methacrylate alkene at 5.8 and 6.2 ppm to the integrals originating from the protons of HA backbones.

### 2.3. Short-Chain HA Loading and Hydrogel Formation

Short-chain hyaluronic acid (3 kDa HA or sHA) was physically added at various concentrations (Table 1) into 3% *w*/*v* of methacrylated HA (MeHA) solution. Dithiothreitol (DTT) was added into the pre-gel solution at a molar ratio of thiol:ene = 2:1, then pH was adjusted in the range of 9–11 to form a gel.

### 2.4. Gel Point Determination

Gelation time of hydrogels was detected by inverted tube methods and oscillation time sweep. First, the tube containing 0.5 mL of mixed pre-gel solution, prepared as previously described, was inverted. The gelation time was defined as the time that the liquid transformed to gel (each sample showed no flow within 20 s during tube inversion). The gelation time was confirmed by oscillation time sweep mode using HAAKE^TM^ MARS^TM^ rheometer (Thermo Scientific, Karlsruhe, Germany). The pre-gel solution was placed between 35 mm of titanium parallel plate and rotor, with 1% strain and a frequency of 1 Hz at 25 °C. The gelation time was decided from where the storage modulus (G′) was equal to the loss modulus (G″) (cross-over point), which represents liquid–solid transition.

### 2.5. Mechanical Property Analysis

HAAKE^TM^ MARS^TM^ rheometer (Thermo Scientific, Karlsruhe, Germany) was used to analyze the rheological properties of the hydrogels. One milliliter samples were placed between the 35 mm of titanium parallel plate and rotor. After that, samples were analyzed under oscillation amplitude sweep mode (1–1000% strain and a frequency of 1 Hz at 25 °C). The mechanical profile of each formulation was reported, as well as the yield point value, which was plotted from the relationship between tan δ and percentage of strain. Tan δ was calculated following this equation:

Tan δ = G″/G′

### 2.6. Swelling and Degradation Behavior

The gel disks (1 mm thickness, 8 mm diameter) were punched from the silicone mold before transferred to centrifuge tubes, weighed, then immersed in 3 mL of PBS for 48 h. At each time point, the swollen gels were weighed again. The swelling ratio was reported, which was calculated from the following equation:

Swelling ratio = (µ_t_ − µ_0_)/µ_0_,

where µ_t_ refers to the weight of the swollen gel at each time point, and µ_0_ is the initial weight of the gel sample.

The degradation study was performed in PBS for 21 days. At each time point, the resting gels were washed with distilled water before being lyophilized for 2 days and weighed. The degradation ratio was calculated as the below equation:

Weight loss ratio = (µ_0_ − µ_d_)/µ_d_, (1)

where µ_0_ and µ_d_ are the initial weight of gel sample and the weight of dry gel sample at each time point, respectively [16].

### 2.7. Micromorphology Analysis

The punched gels (1 mm thickness, 8 mm diameter) were frozen in liquid nitrogen for 5–30 min before being lyophilized for 48 h. Sputter films were coated on the materials for Scanning Electron Microscope (SEM (IT-500HR), JEOL, Tokyo, Japan). Surfaces and internal sections of each lyophilized hydrogel were observed at 3 random locations per sample. The pore size was measured by Image-Analysis J 1.45S software.

### 2.8. Short-Chain HA Released Kinetics

The release kinetics of 3 kDa HA-loaded hydrogels were evaluated in PBS (pH 7.0) for 48 h. The hydrogels (8 mm thickness, 13 mm diameter) were transferred to dialysis bags (12–14 kDa (MWCO), Spectra/Por^®^ 4, Spectrum Laboratories, New Brighton, MN, USA), immersed in 100 mL of PBS, and incubated at 37 °C. One milliliter of the solutions were sampled at each time point and replaced with an equal amount of fresh medium. Then, 100 µL of the collected samples were digested with 50 µL of 100 U hyaluronidase and incubated overnight at 37 °C before measuring the amount of released uronic acid by carbazole assay. Briefly, 100 µL of sodium tetraborate in sulfuric acid was added into each tested sample and heated at 100 ± 5 °C for 10 min before being cooled down at room temperature for 15 min. Then, 0.25% carbazole in 50 µL absolute ethanol was added into each tube and heated at 100 ± 5 °C for 10 min. Following this, 100 µL of final solution was transferred to a 96-well plate, after being cooled down at room temperature for 15 min, and analyzed by microplate reader (CLARIOstar^®^, BMG LABTECH, Rotenberg, Germany) at a wavelength of 550 nm. The standard curve of D-glucuronic acid was used to convert the measured absorbances to the concentration of released uronic acid [16].

### 2.9. Cell Cytocompatibility

PDLs were extracted and cultured as the previous protocol [17]. The isolation and culture protocols were approved by the Ethic Committee, Faculty of Dentistry, Chulalongkorn University (No. HREC-DCU 2021-084). Cells at passage 4–14 were used in the in vitro cell studies.

Indirect cytotoxicity assay was performed following ISO 10993-1:2009 (ISO, 2009a) with slight modification, and the hydrogel samples were described as ISO 10993-5:2009 (ISO, 2012) [18,19]. In brief, pre-gel solution was prepared under sterile conditions. After that, the gel was transferred to a sterile silicone mold. The gel was incubated at 37 °C overnight to ensure complete hydrogel formation. Afterwards, the hydrogels were punched into a disk shape with calculated surface area of 3.14 cm^2^. Each hydrogel disc was immersed in 1 mL of serum-free DMEM containing penicillin/streptomycin for 24 h at 37 °C. On the same day, 1 × 10^4^ of PDLs were seeded into each well of a 96-well plate and incubated at 37 °C in a humidified atmosphere supplemented with 5% CO_2_ for 24 h. The following day, the sample extracts (prepared at different concentrations of 12.5%, 25%, 50%, and 100%) were used to replace the cell culture media and incubated at 37 °C for 24 h. In this assay, the extract vehicle, serum-free DMEM, was used as a control. The morphology of the cells was observed the following day by microscopy (Nikon Eclipse TS2, Nikon, Tokyo, Japan). Then, 50 μL of 0.5 mg/mL MTT solution was prepared and added into each well after the medium was removed, and the cells were washed once with PBS. After incubation for 30 min at 37 °C, 100 μL of DMSO replaced the MTT solution to the wells for formazan extraction before being measured by a microplate reader at 570 nm [20].

### 2.10. Proliferation Assay

Sterile MeHA polymers were dissolved in complete DMEM media (DMEM mixing with 10% FBS, 1% L-glutamine, and 1% penicillin–streptomycin) as a pre-gel solution, before being combined with sHA at each concentration in order to form a gel coated layer on 96-well plates. Five thousand cells per 100 µL were seeded on the top of the gel layer and cultured in 5% CO_2_ incubator at 37 °C for 1, 3, 5, and 7 days. At each time-point, the morphology of cells was observed by using phase contrast imaging with microscope (Nikon Eclipse TS2, Nikon, Tokyo, Japan). Additionally, 10% resazurin-based solution, a cell viability indicator, was reacted with the cells at 37 °C for 1 h, before being analyzed with a microplate reader at excitation/emission of 560 ± 15/590 ± 15 nm [21].

### 2.11. Transwell Migration Assay

Twenty-four-well size transwell inserts with an 8.0 µm pore polycarbonate membrane and 0.33 cm^2^ effective growth area (Corning^®^, Corning, NY, USA) were used to perform cell migration experiments. The lower wells were layered with hydrogel and topped up with serum-free media. PDLs were trypsinized and re-suspended in serum-free media, then seeded in each insert at a concentration of 30,000 cells/well. Each seeded insert was placed into each lower well and incubated at 37 °C for 24 h in order to prevent the effects of cell migration. After the maturity period, the cells were fixed with 4% paraformaldehyde for 20 min, permeabilized with acetic acid:methanol at the ratio of 1:3 for 5 min, then stained with 0.7% crystal violet and washed with PBS. A cotton swab was used to remove non-migrated cells from the upper surface of the membrane. The migrated cells were observed by microscope (Nikon Eclipse TS2, Nikon, Tokyo, Japan). Image-Analysis J 1.45S software was used to count the number of migrated cells from five randomly chosen fields (×40) [22].

### 2.12. Animal Study

Male Wistar rats (250–300 g) were obtained from the Nomura Siam International Co. Ltd. All the in vivo studies were approved by the Faculty of Pharmaceutical Sciences Animal Care and Use Committee, Chulalongkorn University, protocol number 1933004. The housing and experimental procedures complied with the guidelines of the Animals for Scientific Purposes Act, B.E. 2558 (A.D. 2015), Thailand. Prior to the beginning of in vivo experiments, animals were quarantined/acclimated for 7 days to adjust to the standardized laboratory temperature (22 ± 2 °C), humidity (40–60%), and light–dark cycle (12:12 h).

In total, 36 animals were used (calculated using G*Power program version 3.1.9.4) and were randomly divided into three groups with *n* = 12 per group: Control, MeHA, and 1.5% *w*/*v* sHA. The randomization was generated from the random function (Rand) in Excel program. On day 0, the rats were anesthetized using isoflurane (1–3%) with 100% oxygen. The depth of anesthesia and vital signs of the animals were monitored every 5 min. Subcutaneous injection of tramadol was used for peri-operative and post-operative analgesia. Moreover, moist feed was provided at the floor cage level to promote the eating of the rats after the procedure. The oral wounds were created by 3-mm diameter punch biopsy on the anterior palate in the mucoperiosteum of the midline of the hard palate. The soft tissue was removed by sharp dissection. Cotton gauze was placed over the wound until hemostasis was achieved. All procedures were performed using an aseptic technique. The treatment gels were applied to the wound. Every 1, 3, 5, and 7 days after treatment, 3 rats from each group were euthanized by CO_2_ asphyxiation, following the open thorax (American Veterinary Medical Association (AVMA) Guidelines for Euthanasia (2013 edition). The hard palate samples were collected for histopathological studies. The wound sizes were measured. For the histological analysis, the sample sections were stained with Hematoxylin and Eosin (H&E) staining and observed by Apotome.2 apparatus (Carl Zeiss, Jena, Germany) (×400) [23].

### 2.13. Statistical Analysis

All results were reported as mean ± standard deviation (SD), resulting from three or more replications. Statistical significance between different groups was analyzed using one-way analysis of variance followed by Tukey’s post hoc test using GraphPad Prism Software (V.5) with a *p*-value < 0.05.

## 3. Results

### 3.1. HA Modification and Hydrogels Fabrication

**Figure 1:**
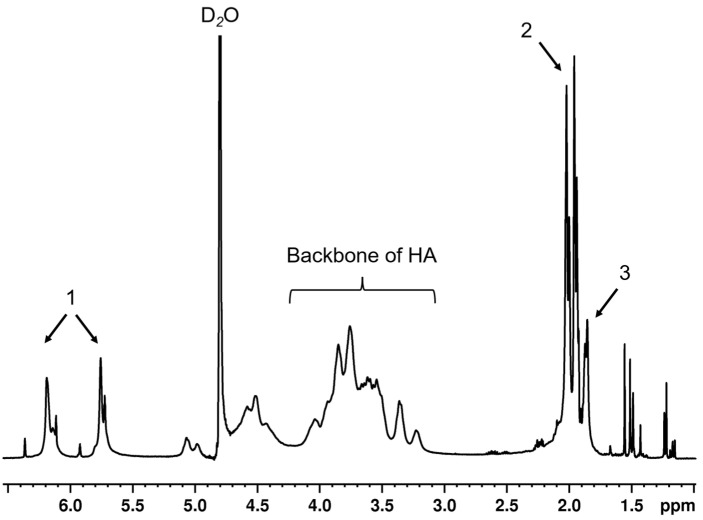
^1^H NMR spectra of methacrylated hyaluronic acid with 80–100% degrees of grafting. The peaks no. 1, 2, and 3 represent 2 protons on methacrylate alkene, 3 protons of the methyl group on the methacrylate, and another 3 protons of the methyl group of HA.

HA polymers were successfully modified at the primary alcohol with methacrylates and presented ~80–100% modifications, analyzed by ^1^H NMR technique (Figure 1). To evaluate the effects of sHA at each concentration on the physicochemical and biological properties of base MeHA hydrogels system, sHA was physically added into the pre-gel solution of MeHA macromers before covalently crosslinking via a Michael addition reaction using a thiol group-containing crosslinker, dithiothreitol (DTT). All hydrogel systems were successfully fabricated and were used for the tests in further studies.

### 3.2. Gel Point Determination

As the aim of this study was to develop an *in situ* forming hydrogel, the gelation time should be tested. The gelation time of hydrogels was observed by the inverted tubes method at room temperature, which demonstrated the gelling behavior of network systems in practical use. The results showed that the gelation time of the hydrogel formulations with or without sHA was around 2–3 min and did not show any significant difference between different formulations. The gel points were also clarified by rheology profile (performed at 25 °C), which determined the crossover point of G″ and G′ during the transition of sol to gel state. The results exhibited that the crossover points were around 1–2 min. The presence of medium and high concentration of sHA in the hydrogel showed faster gelation time (~1 min), while at a low concentration, appeared to have similar gel time with the base MeHA hydrogel. Despite the small variation in gelation times there were no statistically significant differences observed between all groups (Table 2).

### 3.3. Mechanical Property of Hydrogels

**Figure 2:**
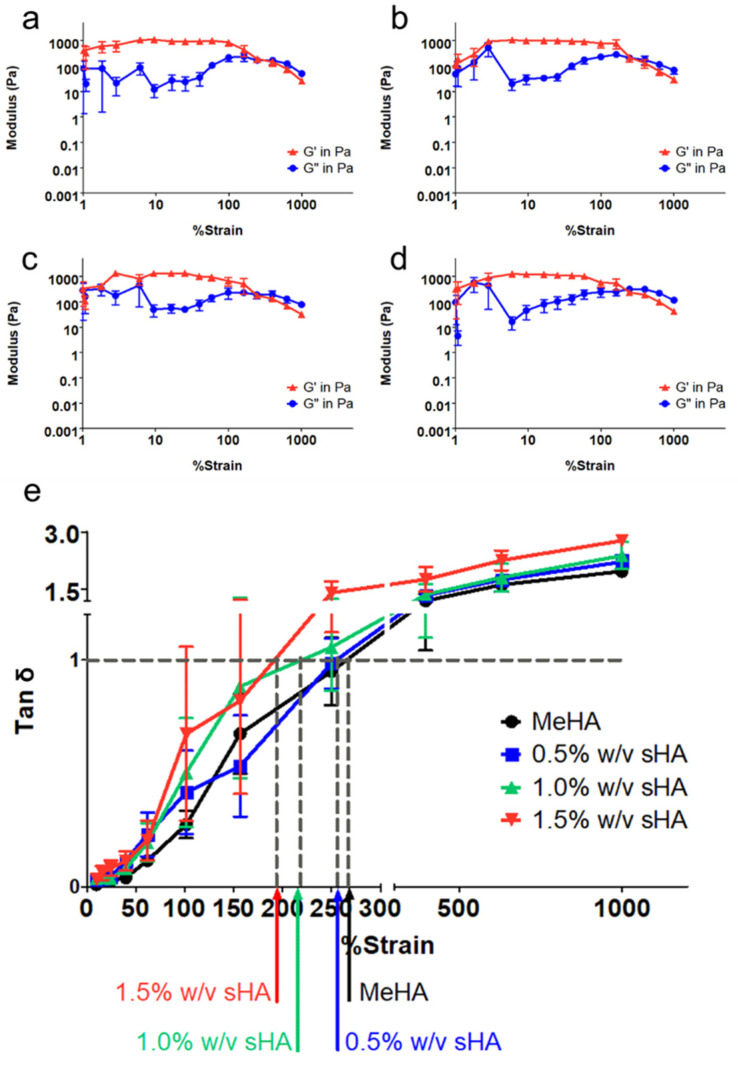
Mechanical profiles of hydrogels, (**a**–**d**) show the mechanical profiles of hydrogel formulations containing 0.0%, 0.5%, 1.0%, and 1.5% *w*/*v* of sHA, respectively. The relationship between tan δ and % strain is shown as in (**e**).

Rheological analysis quantitatively estimated the elastic responses of the MeHA-based hydrogel by simulated giving an external strain ranking from 1–1000% to the gel. Figure 2a–d showed the mechanical profile of each hydrogel formulation using amplitude sweep mode. The storage modulus (G′) of four types of gel were in the range of 1000–1500 Pa, as shown in Table 3. Given % strain above 100, the networks became ruptured. Tan delta (δ), calculated from G″/G′, plotted versus strain of investigated materials are shown in Figure 2e. The intersections of the horizontal line across the graph (where tan δ = 1 is the origin) are known as yield points, which represent the maximum strain that the materials could tolerate. The yield point decreased with an increase in the amount of sHA. 

### 3.4. Swelling and Degradation Behavior

**Figure 3:**
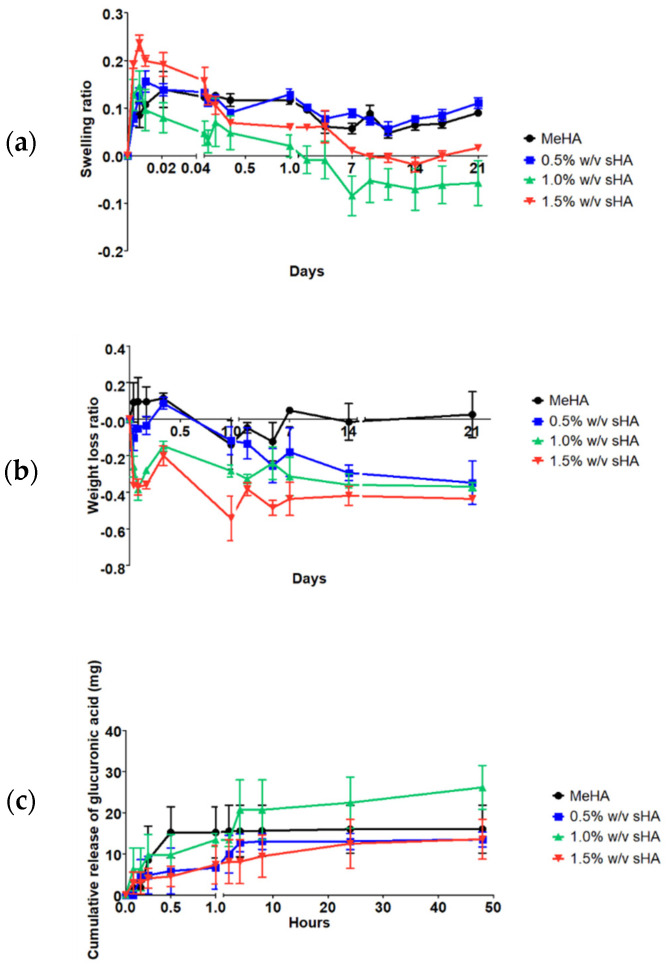
Physicochemical profiles of hydrogels including (**a**) swelling profiles (for 21 days), (**b**) degradation profiles (for 21 days), and (**c**) glucuronic acid release profiles for 2 days.

The swelling ratio across time of four formulations was reported in Figure 3a. The maximum swelling ratio of hydrogels occurred within 1 h. The hydrogel containing a high content of sHA (1.5% *w*/*v* sHA) exhibited the largest swelling ratio (~0.24), while the other formulations were in the range of ~0.14–0.16. The swollen hydrogels reached equilibrium after being immersed in PBS for 7 days. We found that the overall swelling profile of the hydrogel with the low content of sHA was similar to the base MeHA hydrogel. It was noticeable that the medium amount of sHA hydrogel presented the lowest swelling ratio and started to degrade faster than other formulations. Though the hydrogel loaded with high concentration of sHA had the highest swelling ratio at the initial time point, later we also observed a degradation trend. From this study, we can conclude that having a high content of sHA would increase the swelling ratio of the networks. To confirm the effect of sHA on the hydrogel’s degradation, the degradation test was performed in PBS at 37 °C for 21 days, as shown in Figure 3b. The base MeHA hydrogel was the only formula that exhibited a stable weight loss ratio. The higher the content of sHA, the greater the degradation ratio that occurred.

### 3.5. In Vitro Release Study of sHA

Figure 3c exhibited the release profile of sHA from the modified HA hydrogel networks performed under a controlled temperature of 37 °C for 48 h. In this study, we found that, even in the base MeHA hydrogel, some free part of HA from the MeHA scaffold was released in the medium. Some amounts of glucuronic acid were detected, which were generated by the hyaluronidase enzyme digestion prior to the carbazole assay. For the three sHA incorporated hydrogels, there is no sHA concentration-dependent activity regarding the release profile of glucuronic acid. We also found the correlation between release profile and swelling behavior of the hydrogels. The hydrogel containing of 1.0% *w*/*v* of sHA presented the highest released amount of glucuronic acid (~26 mg after soaking in PBS for 48 h or ~65% of total solid mass).

### 3.6. Microstructure of Hydrogels

**Figure 4:**
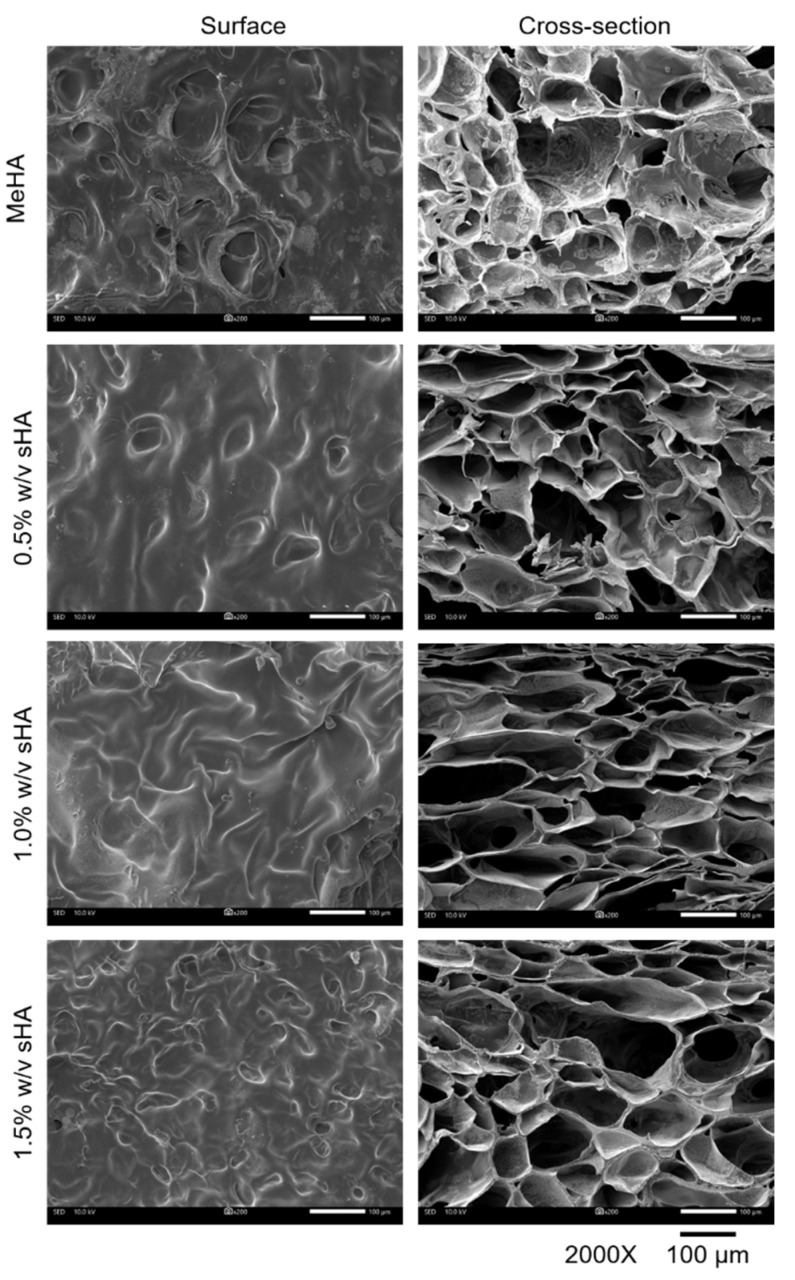
Microstructure images of freeze-dried hydrogels captured by using SEM technique. The scale bars of 2000× magnification are 100 µm.

The microstructures of all gel types investigated by SEM are shown in Figure 4. The hydrogels in all formulations appeared porous in architecture, which related to their resemblance to a crosslinking system. The high sHA hydrogel generated the smallest pore size (~58 µm) compared to others (~64–66 µm). This SEM result showed that the increase in polymer mass by having interpenetrating sHA could reduce the pore size of hydrogel.

### 3.7. Wound Healing Activity of Hydrogels on Periodontal Ligament Stem Cells (PDLs)

To assess the wound healing properties of MeHA hydrogels loaded with sHA, we first performed in vitro cell studies, including a proliferation assay and migration test with PDLs, because establishing a PDL cell population in a periodontal defect is an important requirement for the remodeling and rebuilding of the periodontium [24].

**Figure 5:**
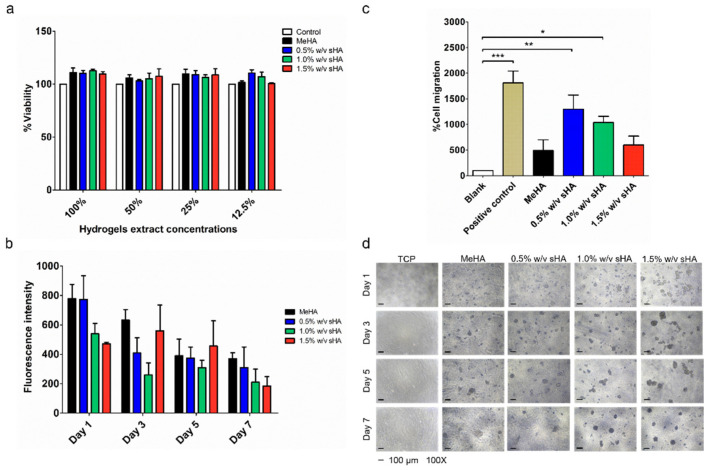
Wound healing activity of hydrogels on PDLs; the hydrogels were tested for their cytocompatibility, indirectly, before being utilized in the other in vitro cell studies (**a**). (**c**) shows the capability of the hydrogels when performing the transwell migration test (for 2 days), serum-free media and serum-contained media were used as a blank and positive control in this study. The significant level of the *p* value was marked with *, ** and *** which refer to *p* value ≤ 0.05, 0.01 and 0.001, respectively. The performance of the hydrogels on promoting the proliferation of PDLs (for 7 days) is shown in (**b**,**d**). (**b**) presents the viability of PDLs, which was indicated and converted into the fluorescence intensity using a resazurin-based solution. (**d**) illustrates the physical appearance of the cells after being cultured on the gels for 7 days.

Prior to evaluating the wound healing activity, the cytocompatibility of hydrogels was performed via indirect cytocompatibility assay. The results indicated that all hydrogel formulations were compatible with PDLs (% cells viability ≥80%) (Figure 5a).

The proliferation test was performed by growing PDLs on the surface of hydrogels to demonstrate their practical use dimension, wherein the cell would directly contact to the surface of hydrogels. Figure 5b illustrated the alterations in the metabolic activities and morphology of the cells during the culture time of 7 days. The results of the metabolic activities, measured by resazurin assay, showed no significant differences among all samples. Cells on hydrogels had lower proliferation rates than cells on tissue culture plates (TCP), with an observable deficiency of cell attachment on the hydrogels’ surfaces. The changes in cell morphology into spheroid shape shown in Figure 5d reflected the low binding behavior. Moreover, we found that extra sHA in the hydrogel formulation showed a lower proliferation rate than the non-sHA formulation.

The transwell migration assay was performed to observe the effects of chemoattractant, released from hydrogels, on PDLs migration. In this study, the cells treated with starving serum and serum containing media were used as a blank and positive control, respectively. The sample hydrogels were layered in the lower well, while the cells were seeded in the insert well. The observed cells were migrated from the upper surface to the lower surface of the polycarbonate membrane of the insert well. The results showed that cells treated with all types of hydrogels had a lower number of migrated cells when compared to the positive control, yet a greater number than the blank. The cells treated with the low content of sHA hydrogel showed the greatest number of migrated cells, following by the medium content of sHA hydrogel. The hydrogel containing the high sHA content exhibited a similar number of migrated cells to the base MeHA hydrogel (Figure 5c).

### 3.8. In Vivo Oral Wound Healing Study

**Figure 6:**
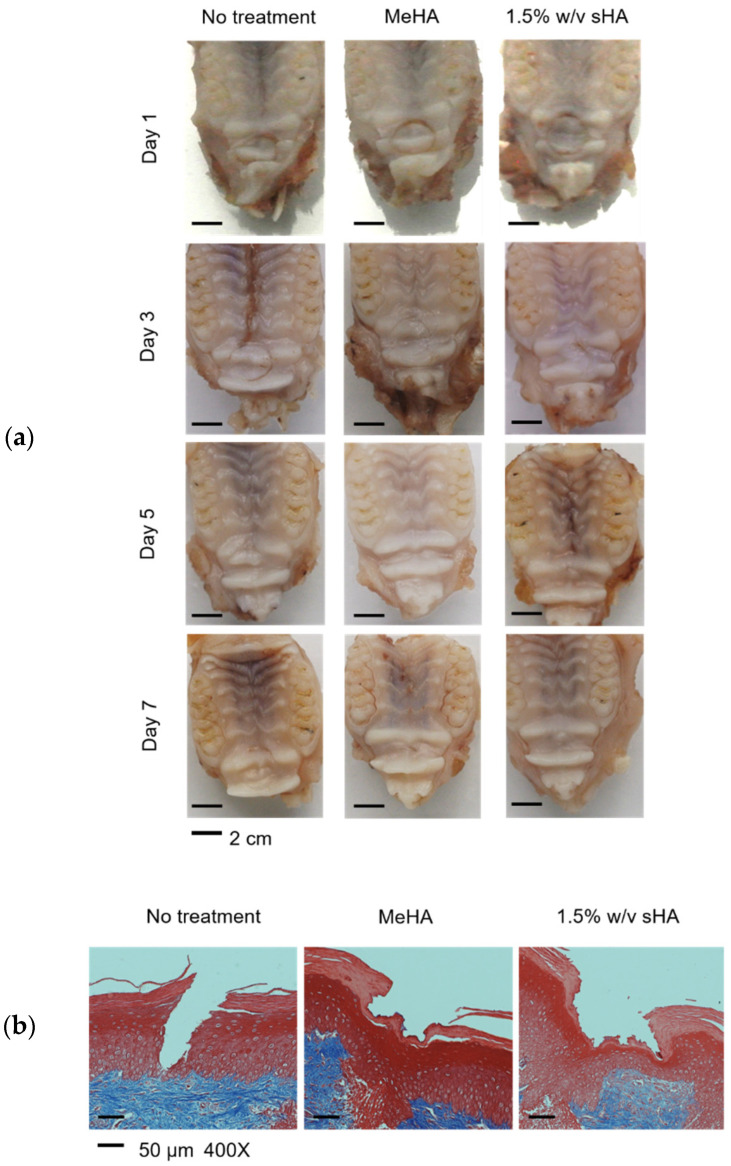
The physical appearance of the wounds on the rats’ palates in three groups of intervention at each time point, with scale bars of 2 cm (**a**). (**b**) shows the histological sections of the rodents palates, staining with hematoxylin and eosin (H&E) after 1 day of treatment, with 50 µm scale bars of 400× magnification.

In order to evaluate the effect of hydrogels on wound healing, the wounds were created on the upper palate of rats by punching. The physical appearance of each sample was captured and is shown in Figure 6a. In this study, we had three conditions to compare; untreated, treated with MeHA hydrogel, and treated with 1.5% *w*/*v* sHA. All wound areas were completely recovered by 7 days. The histological samples indicated that the puncture destroyed two outer layers of the epidermis, including the keratin layer and keratinized stratified squamous epithelium [25]. The results showed that both hydrogel formulations were compatible with the rat oral tissues, with undetectable side effects. Moreover, faster recovery of the epithelium layer (after one day of treatment), when compared to the untreated groups, was observed. Further, the restoration of the keratin layer was also found in both hydrogel groups (Figure 6b). However, we did not find any significant differences between both groups that received hydrogel treatment, due to the limitations of the wound size measurement.

## 4. Discussion

There are numerous clinical HA products that have been approved by the Food and Drug administration for use as medical devices for dental diseases (e.g., Gengigel, Flex Barrier) [26,27]. These products are in gel form, that provides weak mechanical properties and stability, resulting in increased frequency of use. Since the treatment of periodontal disease is long-term management, reducing frequency of use could improve patients’ compliance. In this work, we chose to fabricate the scaffolding of hydrogels from a modified HA chain (MeHA), since this system can be digested and supply HA itself. Moreover, the mechanical properties of this hydrogel can be adjusted by degree of modification or crosslinking system. Another attractive characteristic of this hydrogel system is that it represents itself as an *in situ* gel system, a feature of drug delivery systems that are in liquid forms before administration; once it reaches the target areas, it transforms into a gel [28]. This feature is very useful as it could prevent the deposition of food residues into the depth or irregular shapes of periodontal wounds.

HA fragments have been found mostly in tissue inflammation areas [29,30]. On the other hand, it has been reported that HA fragments could probably enhance the regeneration of damaged tissues [31]. A previous study has revealed that a hydrogel containing two different sizes of HA (long-chain vs. fragmented HA), could lower the inflammatory effect of HA fragments, and improve the attachment and proliferation of endothelial cells. They also found that HA fragments showed some interference with the physical properties of hydrogels (swelling profiles, mechanical properties, crosslinking density) [14]. Trakiatkul and team, illustrated the suitability of a MeHA-based hydrogel for loading polysaccharides and proteins, such as mannitol and BSA. They found no significant changes to the physical properties of this hydrogel system when carrying mannitol/BSA [32]. Accordingly, we decided to explore the possibility of directly incorporating HA fragments (or sHA) into a MeHA injectable hydrogel, and we sought to investigate the impact of sHA content on the gel’s physical properties, in addition to their biological effects (e.g., biocompatibility, migratory).

One of the huge challenges in developing an *in situ* gel system is to obtain an appropriate gelation time. Having a short gelation time is essential for the prompt attachment of the gel to the wound or pockets in oral cavities and to minimize the loss of intra-pocket carriers, further, enhancing patients’ compliance [33]. In order to observe the effects of incorporated sHA on gelation time, the concentration of MeHA and the crosslinking molar ratios (thiol:ene) were fixed at 3% and 2:1, based on our prior work [34]. The content of the incorporated sHA were divided into three concentrations, 0.5%, 1.0%, and 1.5% *w*/*v* (referring to the concentration of commercial products containing HA, which are in the range of 0.2–1.0% *w*/*w*) [27,35]. Our prior work reported the typical gelation time of a hydrogel fabricated from MeHA with 40–60% modifications, which was in the range of 15–30 min [34]. The increase in modification degree minimizes gelation time, due to the increasing of crosslinking moiety (vinyl groups) [36]. In this study, we adjusted the degree of modification of MeHA into the range of 80–100% in order to minimize the gel onset time. From the results, we could achieve gelation time within a few minutes. Additional sHA seemed to have effects on the gelation time, since the gel time of the formulations containing 1.0 and 1.5% *w*/*v* of sHA were less than others, which was related to their pre-gel viscosity, which seemed to be lower than the 0.5% *w*/*v* and non-sHA formulation. Existing HA fragments in an HA macromers solution has been reported to interrupt the interpolymeric interaction of HA solutions [33]. Therefore, a higher content of sHA in a MeHA solution may interfere with the networking system between MeHA macromer chains dissolved in aqueous solutions, resulting in the decrease of viscosity that allowed a faster crosslinking reaction and showed an earlier sol to gel transition. Nonetheless, adding sHA at this range of concentration did not have a significant impact on the gelation time.

Rheological analysis quantitatively estimated the elastic responses of a MeHA-based hydrogel by simulating an external strain ranking from 1–1000% to the gel. By increasing the degree of modification of MeHA, we could obtain the storage modulus (G′) of approximately 1000–1500 Pa; that was even higher than that of a previously developed MeHA hydrogel with 40% degree of modification; the G′ was ~400 Pa [16]. Though sHA did not have an effect on G′, it seemed to have interference with the yield point of hydrogels, which represents the maximum tolerance of the material to given strain. Increasing amounts of sHA reduces the yield point of hydrogels; we believed that this event was related to the possible causes of the high solid contents in the formulation. We observed a correlation between the yield point and microstructure of the hydrogels. A high percentage of sHA incorporation causes a weaker gel structure; additionally, the presence of small pore size might be the effect of entanglement between polymers which formed an impermanent interaction across networks [37]. Another possible reason is the disturbance of sHA to the crosslinking system. Overall, there was no significant difference among all hydrogels, they could tolerate to external strains of 150–300% strain, which could be survivable when surrounded by distributed strain during mastication [38].

In order to evaluate the long-term retention of incorporated sHA within the MeHA hydrogel, a swelling and degradation study, and in vitro release study should be performed. We found that a high content of sHA increased the gel swelling and degradation ratio. This phenomenon was related to a previous study, which reported that the effect of having HA oligomers in an HA hydrogel could increase the swelling ratio and cause low crosslinking density [14]. We also found an association between the release profile and swelling behavior of the hydrogels. The highest released amount of glucuronic acid was found in the 1.0% *w*/*v* of sHA hydrogel, it also had the highest swelling ratio and fastest weight loss, which could refer to the early degradation of the hydrogel formulation. On the other hand, the formulation containing 1.5% *w*/*v* of sHA could slow release the amount of glucuronic acid, which associated to the smaller pore size of itself, compared to the other formulation. All in all, the addition of sHA at these concentrations did not have much interference on the hydrogels’ physical properties.

To assess the wound healing properties of MeHA hydrogels loaded with sHA, we firstly performed in vitro cell studies, including a proliferation assay and migration test with PDLs, because establishing a PDL cell population in a periodontal defect is an important requirement for the remodeling and rebuilding of the periodontium [24]. All hydrogels were proved to be biocompatible with PDLs. We executed the proliferation test by culturing PDLs on hydrogels to demonstrate their practical use dimension, wherein the cell would directly contact to the surface of the hydrogels. We found that PDLS appeared to have poor attachment on the surface of hydrogels, forming a spheroidal shape, which impacts their proliferation behavior. Two factors known to influence this phenomenon are the material stiffness and receptor binding affinity of the cell and modified hyaluronan. Material stiffness plays a vital role in controlling cell behavior. Recently, a study reported that the hardness of culture material (ranking from 6–135 kPa) had effects on the alteration of PDLs’ proliferation rate. PDLs cultured on soft material (6 kPa) showed an inferior proliferation rate [12,39]. Therefore, it can be presumed that the elastic modulus of the hydrogels (around 1–1.5 kPa) would not be strong enough to support cell proliferation. Another related reason is the binding affinity of the cell and modified HA. The hydroxyl groups on the HA chain are used as one of the binding sites that could associate to CD44 [40]. Kwon and coworkers disclosed the relationship between the level of HA modification and binding affinity to CD44. A high level of HA modification had poor binding affinity to CD44 due to the unavailable binding site [41]. Since the investigated MeHA had very high degree of modification, this could cause the deficiency of HA-CD44 binding which resulted in poor cell-surface attachment.

For the transwell migration test, we found that the hydrogel containing 0.5% *w*/*v* of sHA presented the best performance in inducing migration. We hypothesized that some amount of sHA would have a chemo-attractive effect. This assumption is in agreement with an earlier study, which found that HA promotes the migration of human meniscus cells in a concentration-dependent manner [42]. On the contrary, the result of this study showed that the increased amount of sHA resulted in the decrease of migrated cells. The possible reason for this is that the release of sHA from the highest content of sHA hydrogel formulation was hindered by the small pore size of the hydrogels. The microstructure study mentioned above found that the pore size of the gels was reduced as a consequence of an increase in the content of sHA in the network.

As the hydrogel formulation containing 1.5% *w*/*v* of sHA appeared to have a prolonged release effect of sHA, it was selected for further study of its impacts on tissue regeneration in an in vivo model, while the base MeHA gel was utilized as a blank. Small defects were simulated on the rats’ upper palates by punching, which damaged the epidermis layers (keratin layer and keratinized stratified squamous epithelium) [25]. Our findings showed that applying hydrogels to the wound site reduced the treatment consuming time. In histological section, our hydrogels could induce epithelium cells originating from the three basal layers of the oral squamous epithelium which migrated and repaired the injury caused by the shallower depth of the wound [43]. Our work has proved the possibility of forming two different sizes of HA and clarified the interference with the physical properties of the hydrogel, which did not show any significant changes. Our study illustrated that our hydrogels were biocompatible with tissues in the oral cavity, in both in vitro and in vivo models. The ability to shorten the healing time of the hydrogels could promote the chance to use this approach as a medical device to support the regeneration of damaged tissues related to dental diseases; however, further studies are needed.

## 5. Conclusions

In summary, this work provided evidence that supports the possibility of applying an injectable HA-based hydrogel approach to dental tissue regeneration. This system could contribute an optimal gelation time for practical use. Physical incorporation of sHA could minimally alter the networking system. The overall physicochemical properties of all types of hydrogels were not significantly different. Therefore, the MeHA hydrogel could be a suitable delivery system for low molecular weight macromolecules of approximately the same molecular weight as the sHA used in this study. Moreover, all hydrogels were biocompatible with PDLs. sHA showed chemo-attractive activity, with the 0.5% *w*/*v* sHA treated in transwell culture of PDLs exhibiting the greater migratory activity. The interpenetration of sHA into the hydrogel scaffolding network was assumed to be the cause of the lower amount of free sHA released from the scaffold. However, the hydrogel containing a high concentration of sHA and the non-sHA hydrogel showed good benefits by providing faster restoration of damaged epithelial tissue (in vivo model). These results encourage further development and investigation of hyaluronic acid-based hydrogels in periodontal regeneration.

## Figures and Tables

**Table 1:** Concentration of sHA in hydrogel.

Hydrogel Formulations (Prefix Name)	Concentration of sHA (% *w*/*v*)
MeHA	0
0.5% *w*/*v* sHA	0.5
1.0% *w*/*v* sHA	1.0
1.5% *w*/*v* sHA	1.5

**Table 2:** The gelation time of four types of hydrogel formulations, detected by inverted tube method and by rheology profile where G′ = G″.

Formulation	Gelation Time (Minute)	
	Inverted Tube Method	G′ = G″
MeHA	2.20 ± 0.60	2.30 ± 1.10
0.5% *w*/*v* sHA	2.50 ± 0.80	3.00 ± 1.40
1.0% *w*/*v* sHA	2.50 ± 0.50	1.00 ± 0.20
1.5% *w*/*v* sHA	3.00 ± 1.20	1.10 ± 0.30

G′: storage modulus. G″: loss modulus. Data are represented as mean ± SD. No statistical significance is represented.

**Table 3:** The mechanical analysis of four types of hydrogel formulations.

Formulations	Storage Modulus G′ (Pa)	Yield Point (tan δ)
MeHA	1210 ± 140	290 ± 160
0.5% *w*/*v* sHA	1120 ± 210	270 ± 80
1.0% *w*/*v* sHA	1480 ± 360	250 ± 170
1.5% *w*/*v* sHA	1300 ± 340	180 ± 70

Data are represented as mean ± SD. No statistical significance is represented.

## Data Availability

Data are available upon request.
